# Use of Robotics in Colorectal Surgery: An Initial Single-Centre Experience

**DOI:** 10.7759/cureus.93786

**Published:** 2025-10-03

**Authors:** Anurag Agarwal, Anil Lala, Sheik Fazal Ur Rehman, Steve Dixon, Graham Whiteley, Sjaak Pouwels, Ahmed Ahmed, Suhaib Ahmad

**Affiliations:** 1 General and Colorectal Surgery, Betsi Cadwaladr University Health Board, Bangor, GBR; 2 General and Colorectal Surgery, Glan Clwyd Hospital, Rhyl, GBR; 3 General and Colorectal Surgery, Betsi Cadwaladr University Health Board, Wrexham, GBR; 4 Intensive Care Medicine, Elisabeth-Tweesteden Hospital, Tilburg, NLD; 5 General Surgery, Imperial College London, London, GBR; 6 General and Colorectal Surgery, Health Education and Improvement Wales (HEIW), Bangor, GBR

**Keywords:** colon malignancies, colorectal cancer, general surgery, laparoscopic colon resection, laparoscopic surgery, minimal access surgery, robot-assisted surgery

## Abstract

Background

Robotic-assisted surgery is increasingly recognised as a viable alternative to conventional open and laparoscopic techniques in colorectal cancer (CRC) management. This study presents a single-centre initial experience with the Versius® robotic system for colorectal resections between April 2023 and March 2024.

Methodology

A retrospective cohort study was conducted at a University Health Board in the United Kingdom, involving 56 patients who underwent robotic-assisted colorectal resections for CRC. Patients aged ≥18 years with radiologically and endoscopically confirmed non-metastatic CRC (American Joint Committee on Cancer stage T1-4, N0-2, M0) were included. Data on preoperative, intraoperative, and postoperative parameters were collected. Operative details included procedure type, console time, and estimated blood loss. Complications were graded using the Clavien-Dindo classification. Analyses were performed using SPSS version 26 (IBM Corp., Armonk, NY, USA). Categorical variables were reported as frequencies and percentages; continuous variables as means ± SD or medians with interquartile ranges. Chi-square, independent t-tests, or Mann-Whitney U tests were used for group comparisons. A p-value <0.05 was considered significant.

Results

The mean operative time was 131 ± 50.99 minutes. In-hospital complications occurred in 37.5% of patients, predominantly local (26.8%) over systemic (10.7%). No significant association was found between preoperative variables and postoperative complications. No perioperative mortality occurred.

Conclusions

The Versius® system demonstrates feasibility and safety for CRC resections, with acceptable complication rates and no mortality, supporting its use as a promising approach in robotic colorectal surgery.

## Introduction

Colorectal cancer (CRC) is a malignant condition affecting the colon (large intestine) and rectum. According to the World Health Organization (WHO), with enhanced monitoring in high-income countries, CRC incidence rates have been falling [1]. In 2020, over 1.9 million new cases and more than 930,000 deaths from CRC were reported globally [1,2]. By 2040, it is projected that the number of new CRC cases will rise to 3.2 million annually, marking a 63% increase, and the number of deaths will reach 1.6 million per year, representing a 73% rise [1]. The incidence of CRC rises markedly after age 50, and risk is further influenced by lifestyle factors such as tobacco and alcohol use, physical inactivity, and dietary patterns. A family history of CRC is also an established risk factor [3,4].

Surgical resection remains the primary treatment for early-stage CRC. In certain cases, neoadjuvant chemotherapy (before surgery) may be administered to reduce the size of the tumour. Radiation therapy is often used for rectal tumours to enhance the likelihood of shrinking the tumour [5,6]. Adjuvant chemotherapy, on the other hand, may be recommended to target any residual cancer cells not visible or removable during surgery. With advancements in medical technology, minimally invasive surgery (MIS) is increasingly replacing traditional open procedures due to its improved outcomes. The advent of robotic surgical devices has further enhanced the perioperative experience for surgeons, offering benefits such as improved three-dimensional visualisation of the surgical area, better communication with the surgical team, and versatility in use across different operating rooms [7].

The Versius® robotic system (CMR Surgical) is a modular platform that mimics the human arm, offering flexible port placement, a 710° range of motion, and three-dimensional visualisation to improve precision and ergonomics in minimally invasive colorectal surgery [8]. Clinical studies have demonstrated feasibility and safety, including favourable outcomes in patients undergoing low anterior resection without positive circumferential margins [9], and the system has been adopted within the UK National Health Service for colorectal procedures [10]. By enhancing visualisation and dexterity in confined pelvic spaces, particularly for rectal carcinoma [7,11], Versius® supports the global transition toward MIS, which is associated with reduced pain, shorter hospital stay, and faster recovery compared with open procedures.

The primary objective of this study was to evaluate the initial experience of CRC resections performed with the Versius® robotic system, focusing on perioperative outcomes and complication rates. Secondary objectives included assessing the inflammatory response and patient recovery during hospitalisation and after discharge, as well as contextualising these findings within the broader surgical landscape to highlight the strengths and limitations of this approach.

## Materials and methods

Study design

This was a retrospective cohort study of 56 patients diagnosed with CRC who underwent robotic-assisted surgery between April 2023 and March 2024 at a University Health Board in the United Kingdom. The study adhered to the principles outlined in the STROBE reporting guideline and received ethical approval from the Institutional Review Board (approval number: 349170).

Patient recruitment

Patients were eligible for inclusion if they were aged 18 years or older, had a radiologically and endoscopically confirmed diagnosis of colonic or rectal carcinoma, and had postoperative histological confirmation of tumour staging as T1-T4, N0-N2, M0 according to the 8th edition of the American Joint Committee on Cancer TNM staging system [12]. Patients who had undergone neoadjuvant chemotherapy or radiotherapy were also included. Exclusion criteria comprised the presence of metastatic disease (M1) at presentation, severe comorbidities, prior major abdominal surgeries, morbid obesity with a body mass index (BMI) greater than 40 kg/m², conversion to open surgery, inability to achieve adequate pneumoperitoneum, tumours requiring multivisceral resection, incomplete patient records, or loss to follow-up before key postoperative assessments.

Training and surgical procedures

The introduction of CMR Versus®-assisted colorectal surgery followed a structured and collaborative training methodology. Surgeons and scrub staff initially completed online modules, practical console training, and, in some cases, virtual reality-based simulations. Each surgeon was required to undergo a mandatory metrics-based practical console assessment before participating in a three-day accredited cadaveric training course alongside four scrub nurses. During the early learning phase, surgeons operated in pairs under mentorship, supported by trained scrub nurses, and each completed three proctored cases. The wider theatre team, including anaesthetic and Pre-Operative Assessment Clinic staff, attended awareness sessions tailored to patient selection and procedural requirements.

All 56 patients underwent robotic-assisted rather than totally robotic surgery. For left-sided colectomies, including sigmoid colectomies and rectal resections, an intracorporeal anastomosis was performed. In contrast, right-sided colectomies were completed with extracorporeal anastomosis. Intracorporeal anastomosis was performed entirely within the abdominal cavity, typically using stapling techniques, offering benefits such as smaller extraction incisions and reduced bowel handling. Extracorporeal anastomosis, performed through a midline or Pfannenstiel incision, was preferred in right-sided colectomies due to shorter intracorporeal operative time and ease of specimen extraction. The choice of anastomotic technique was determined by anatomical considerations and surgeon preference, with the aim of optimising recovery and patient outcomes.

Data collection

Data were collected from electronic health records and operative notes. Demographic variables included age and sex. Operative parameters encompassed total console time in minutes, estimated blood loss, type of colorectal surgery performed (right colectomy, left colectomy, sigmoid colectomy, or rectal resection), and whether patients received neoadjuvant therapy. Tumour staging was assessed radiologically and pathologically. In-hospital outcomes included time to first bowel movement, postoperative pain score was recorded using the Visual Analogue Scale [13], and inflammatory markers such as white cell count, C-reactive protein, serum albumin, estimated glomerular filtration rate, and serum creatinine. Complications were categorised as local or systemic and recorded according to the Clavien-Dindo classification system [14]. Local complications included adverse events at or near the surgical site, such as surgical site infection, anastomotic leak, bleeding, stoma-related issues, or wound complications. Systemic complications refer to physiological disturbances affecting organs beyond the surgical site. Out-of-hospital complications, including 30- and 90-day morbidity and mortality, were also documented.

Statistical analysis

All statistical analyses were conducted using SPSS version 26.0 (IBM Corp., Armonk, NY, USA). Categorical variables were summarised as frequencies and percentages, while continuous variables were first assessed for normality and presented as mean with standard deviation for normally distributed data or median with interquartile range for non-normally distributed data. Associations between categorical variables and postoperative complications were analysed using the chi-square test. For continuous variables, independent t-tests were applied to normally distributed data, while Mann-Whitney U tests were used for non-normally distributed variables. A two-tailed p-value of less than 0.05 was considered statistically significant.

## Results

A total of 56 patients underwent robotic-assisted colorectal surgery. The most common procedures performed were low anterior resection in 18 patients, followed by sigmoid colectomy in 17 patients, abdominoperineal resection in eight patients, right hemicolectomy in seven patients, extended right hemicolectomy in two patients, pan-proctocolectomy in two patients, subtotal colectomy in one patient, and left hemicolectomy in one patient. The study group demographics, operative parameters, radiological and pathological results, and in-hospital and out-of-hospital outcomes are shown in Table 1. The mean operative time was 131 ± 50.99 minutes.

**Table 1:** Clinical, pathological, and surgical outcomes. Values in parentheses indicate percentages. APR = abdominoperineal resection; LAR = low anterior resection; Right Hemi = right hemicolectomy; Extended right = extended right hemicolectomy; Panprocto = panproctocolectomy; Left Hemi = left hemicolectomy; Extended left hemi = extended right hemicolectomy; Transverse = transverse colectomy

Variables	Total (n = 56)
Patient demographics	
Age (years)	63.39 ± 12.075
Gender (male/female), n (%)	31 (55.36)/25 (44.64)
Operative parameters	
Console time (minutes)	131 ± 50.99
Estimated blood loss (<50/50–500/>500 mL), n (%)	52 (92.9)/03 (5.4)/01 (1.8)
Type of colorectal surgery, n (APR/LAR/Sigmoid colectomy/Right hemi-/Extended right-/Panprocto-/Subtotal colectomy/Left hemi-/Extended left hemi-/Transverse-)	08/18/17/07/02/02/01/00/01
Neoadjuvant therapy, n (Radio-/Chemoradio-/None)	01/05/50
Radiology results	
T stage, n (x/1/2/3/4)	05/03/21/23/02
N stage, n (0/1/2)	39/13/02
M stage, n (0/1)	54/00
Benign, n	02
Pathology results	
Height of rectal tumour from anal verge (cm)	3.73 ± 5.45
Benign without prior neoadjuvant therapy, n	5
Benign with prior neoadjuvant therapy, n	1
pT stage without prior neoadjuvant therapy, n (T1/T2/T3/T4a/T4b)	07/17/20/01/00
ypT stage with prior neoadjuvant therapy, n (T1/T2/T3/T4a/T4b)	00/02/03/00/00
pN stage without prior neoadjuvant therapy, n (0/1a/1b/1c/2a/2b)	35/06/01/01/02/00
pN stage with prior neoadjuvant therapy, n (0/1a/1b/1c/2a/2b)	04/00/01/00/00/00
pM stage without prior neoadjuvant therapy, n (0/1a/1b/1c)	45/00/00/00
pM stage without prior neoadjuvant therapy, n (0/1a/1b/1c)	05/00/00/00
R stage, n (0/1)	50/01
With positive resection margin, n (%)	01 (1.8)
With Lymphovascular invasion, n (%)	02 (3.6)
In-hospital outcomes	
Time to first bowel movement, (d)	2.34 ± 1.1
Overall complications, n (%)	21 (37.5)
Local complications, n (%)	15 (26.78)
Systemic complications, n (%)	06 (10.7)
Postoperative hospital stay (d)	7.04 ± 3.46
Postoperative pain score	4.77 ± 2.2
Mortality, n (%)	0 (0.0)
Out-of-hospital outcomes	
Postoperative 30 days with complications, n (%)	11 (19.64)
Postoperative 90 days with complications, n (%)	02 (3.6)
Mortality (30 days, 90 days), n	00/00

In-hospital outcomes

Among the 56 patients, the overall in-hospital complication rate was 37.5% (n = 21). Local complications occurred in 15 (26.8%) patients, with surgical site infection being the most frequent, followed by stoma-related issues, ileus, small bowel obstruction, and intra-abdominal bleeding. According to the Clavien-Dindo (C-D) classification, 11 (19.6%) patients experienced Grade I complications, one (1.8%) patient experienced a Grade II complication, two (3.6%) patients experienced Grade IIIa complications, and one (1.8%) patient experienced a Grade IIIb complication. One patient required re-operation due to intra-abdominal bleeding. Systemic complications were documented in six (10.7%) patients, all classified as Grade I. The in-hospital mortality rate was 0%. The detailed distribution of local and systemic complications, stratified by the C-D classification, is summarised in Table 2.

**Table 2:** Clavien-Dindo classification of in-hospital complications (n = 56). This table presents the distribution of local and systemic complications observed during the index admission for robotic-assisted colorectal cancer surgery. Complications are categorised according to the Clavien-Dindo classification system (Grades I–V) [13]. Values represent the number of patients with each complication, with percentages in parentheses. The classification reflects increasing severity, ranging from Grade I (minor complications not requiring intervention) to Grade V (death). No Grade IV or V complications were observed in this study. Values in parentheses indicate percentages.

Complication type	Clavien-Dindo grade	n (%)
Local complications		
Wound problems	Grade I	7 (12.5)
Stoma-related	Grade I	1 (1.78)
	Grade II	1 (1.78)
	Grade IIIa	1 (1.78)
Ileus/Motility-related	Grade I	3 (5.36)
Small bowel obstruction	Grade IIIa	1 (1.78)
Intra-abdominal bleeding	Grade IIIb	1 (1.78)
Systemic complications		
Pulmonary	Grade I	1 (1.78)
Renal	Grade I	1 (1.78)
Central nervous	Grade I	1 (1.78)
Hematologic	Grade I	2 (3.57)

In the immediate postoperative period, there was no significant association between local complications and age of patient (p = 0.0751), preoperative radiological stage of the disease (p = 0.7985), postoperative stage of the disease (p = 0.3013), and type of surgery performed (p = 0.5480).

In addition, there was no significant association between systemic complication and age of patient (p = 0.7450), preoperative radiological stage of the disease (p = 0.4136), postoperative stage of the disease (p = 0.4023), and type of surgery performed (p = 0.8197).

Out-of-hospital outcomes

At 30 days postoperatively, the complication rate was 19.6% (n = 11). Local complications included wound problems, stoma-related issues, anastomotic leakage, and intra-abdominal infection/effusion. Systemic complications were limited to infection. Most were Grade I or II events, but three patients experienced Grade IIIa complications (Table 3).

**Table 3:** Clavien-Dindo classification of out-of-hospital complications at 30 days (n = 56). This table details complications that occurred within 30 days following robotic-assisted colorectal cancer surgery. Complications are classified using the Clavien-Dindo grading system [13], with severity increasing from Grade I to Grade V. The table specifies the type of complication and corresponding grade, with values expressed as absolute numbers and percentages of the total cohort.

Complication type	Clavien-Dindo grade	n (%)
Local complications		
Wound problems	Grade I	3 (5.36)
Stoma-related	Grade I	2 (3.57)
Anastomotic leakage	Grade IIIa	3 (5.36)
Intra-abdominal infection/Effusion	Grade I	2 (3.57)
Systemic complications		
Infection	Grade I	1 (1.78)

At 90 days, the overall complication rate declined to 3.6% (n = 2). One (1.78%) patient developed a stoma-related issue and another developed a genitourinary complication (1.78%), both were Grade I complications as per Calvien-Dindo classification system [13]. No deaths occurred at either 30 or 90 days. One patient developed a metastatic lesion in the cervix and was planned for palliative care after multidisciplinary discussion.

Serial measurements of inflammatory markers, serum albumin, and renal function were performed postoperatively to monitor recovery and detect early complications. White cell count showed a gradual decline from postoperative day one to day seven, stabilising near the upper limit of the reference range. C-reactive protein peaked on postoperative day two and remained elevated throughout the first week, reflecting the expected postoperative inflammatory response. Serum albumin remained consistently below the lower limit of the reference range, with progressive decline over time. Serum creatinine and estimated glomerular filtration rate (eGFR) values remained largely stable, indicating preserved renal function. These postoperative trends are summarised in Table 4.

**Table 4:** Postoperative trends of inflammatory, nutritional, and renal function markers following robotic-assisted colorectal cancer surgery (n = 56). This table presents the mean ± standard deviation (SD) values for white cell count (WCC), C-reactive protein (CRP), serum albumin, serum creatinine, and estimated glomerular filtration rate (eGFR) measured on postoperative days 1 through 7. The table provides reference ranges for clinical interpretation. WCC and CRP reflect inflammatory response, albumin indicates nutritional and inflammatory status, while creatinine and eGFR assess renal function. Persistent deviations from reference values may indicate complications.

Parameter	Day 1	Day 2	Day 3	Day 4	Day 5	Day 6	Day 7	Reference range
White cell count (×10⁹/L)	11.08 ± 2.83	10.32 ± 2.66	9.34 ± 3.04	8.41 ± 2.60	7.76 ± 2.13	8.79 ± 3.36	10.27 ± 3.83	4.0–11.0
C-reactive protein (mg/L)	65.52 ± 43.57	136.13 ± 96.13	125.36 ± 98.94	118.44 ± 95.50	110.89 ± 100.32	117.36 ± 11.28	128.95 ± 90.56	<5
Serum albumin (g/L)	29.92 ± 4.68	30.50 ± 4.98	29.44 ± 4.98	29.87 ± 5.27	29.09 ± 5.79	27.93 ± 5.58	26.95 ± 5.46	35–50
Serum creatinine (µmol/L)	75.20 ± 30.27	74.33 ± 31.74	71.70 ± 28.79	70.47 ± 20.53	72.11 ± 30.57	71.96 ± 33.99	71.27 ± 38.65	45–90 (F), 60–110 (M)
eGFR (mL/minute/1.73m²)	79.82 ± 14.99	80.58 ± 14.66	82.58 ± 14.66	81.22 ± 13.36	81.61 ± 15.09	79.57 ± 17.29	79.55 ± 20.01	≥90

## Discussion

The CMR Versius® robotic surgery system was implemented using a hybrid learning methodology, integrating robotic, laparoscopic, and open surgical techniques for a structured transition and optimal patient outcomes. Initially, a hybrid surgical approach allowed for gradual adaptation while leveraging existing expertise. A collaborative training model, involving consultants from multiple sites with varying experience levels, promoted skill standardisation, procedural efficiency, and cross-institutional knowledge exchange. This multi-institutional approach facilitated efficient and scalable system integration, enhancing the adoption and proficiency of robotic-assisted surgery.

The mean console time of 131 ± 50.99 minutes and the in-hospital complication rate of 37.5%, reported in this study, are comparable to early experiences reported in other studies [11]. On the other hand, while the overall morbidity was within acceptable limits, the presence of local complications (26.78%) highlights that even with advanced technology, surgical outcomes remain influenced by multiple patient- and procedure-related factors. Studies have demonstrated that operative duration and other perioperative factors, such as BMI and the lead surgeon’s experience, are critical determinants of postoperative outcomes [11].

Interestingly, the study’s analysis of postoperative complications using the C-D classification revealed that most local complications were of lower grade (Grade 1 or Grade 3a), with very few high-grade complications. This finding is important as it suggests that while minor adverse events may occur, severe complications remain infrequent. This observation is in line with other contemporary studies, such as those by Kirchhoff et al. [15] and Huang et al. [16], who identified that factors including patient age, comorbidities, and specific surgical techniques can modulate the risk of complications. However, the absence of significant associations between common preoperative parameters and postoperative outcomes in this study might be partly attributed to the limited sample size and the inherent selection bias of a single-centre retrospective design.

The incidence of systemic complications was lower (10.7% in-hospital and 3.6% at 90 days), compared to local complications, which is encouraging given that these complications are typically associated with higher morbidity. The low systemic complication rate may also reflect the rigorous preoperative evaluation and multidisciplinary team approach adopted when treating patients with CRC, emphasising the importance of comprehensive perioperative care. Similar conclusions have been drawn in previous research, which underscores that careful patient selection and standardised protocols are crucial in optimising outcomes in robotic colorectal surgery [15,17].

The white cell count shows a gradual decline from an elevated level on day 1 (11.08 ± 2.83) to a lower value by day seven (10.27 ± 3.83). This pattern is indicative of an acute inflammatory response that peaks shortly after surgery and then gradually subsides as the systemic stress diminishes. Similar trajectories are observed in the CRP values, which peak on day two (136.13 ± 96.13) and remain relatively high through days three to seven. CRP is a well-established marker of inflammation, and its elevated levels in the early postoperative period reflect the body’s response to surgical trauma [18,19]. The sustained elevation through the first week may also suggest a continued inflammatory response or early postoperative complications that warrant close clinical observation.

Conversely, the postoperative albumin levels display a gradual decline from 29.92 ± 4.68 on day one to 26.95 ± 5.46 by day seven. Hypoalbuminemia in the postoperative period can be attributed to the acute-phase response, where albumin, a negative acute-phase reactant, decreases due to both increased capillary permeability and a reduction in hepatic synthesis. Such trends are important as they can impact wound healing and overall recovery [20,21].

Renal function, as assessed by serum creatinine and eGFR, remains relatively stable throughout the postoperative period. The creatinine values show only minimal fluctuations, and eGFR levels remain within a narrow range, suggesting that the robotic surgical approach and associated perioperative management did not adversely affect renal function [22].

Overall, the trends in these biochemical markers provide an objective assessment of the systemic response following robotic colorectal surgery. They underscore the expected inflammatory response and its gradual resolution, while also highlighting the importance of nutritional monitoring and renal function preservation in the postoperative period. These findings are consistent with previous studies on surgical stress responses and validate the efficacy of the minimally invasive approach in managing systemic inflammation and supporting recovery [23-25].

From a cost-effectiveness perspective, robotic surgery is associated with longer operative times and higher initial costs, which may limit its widespread adoption despite the clinical advantages. The current study acknowledges that while robotic procedures appear to be safe and feasible, the financial implications remain a significant consideration. Cost-benefit analyses from other centres suggest that the reduction in postoperative complications and shorter recovery periods might eventually offset the higher upfront costs, but further research is needed to substantiate this claim across diverse healthcare settings [7,11].

Limitations

Despite these positive findings, it is essential to acknowledge the limitations of the study. The retrospective design and relatively small cohort restrict the generalisability of the results. Selection bias was introduced by excluding patients with high BMI, metastatic disease, or complex resections. Moreover, the study period covered only a short-term follow-up, which precludes any definitive conclusions regarding long-term oncological outcomes such as recurrence rates or overall survival. In addition, although complication rates were carefully documented using the C-D classification, the reliance on electronic health records introduces the potential for under-reporting of minor events or incomplete capture of follow-up data. Laboratory markers were recorded consistently, but no formal correlation was performed between inflammatory trends and clinical outcomes, which may represent an area for future investigation. Finally, the study focused on short-term perioperative outcomes, and long-term oncological outcomes, such as disease-free and overall survival, were not assessed. These limitations are consistent with those reported in early robotic surgery studies, and they highlight the need for larger, prospective, multi-centre trials to establish the definitive role of robotic systems such as Versius® in the treatment paradigm of CRC.

## Conclusions

In this initial single-centre experience, the CMR Surgical Versius® system was associated with acceptable operative times and a manageable complication profile in CRC surgery. These results suggest that robotic-assisted procedures are feasible within this setting and can be performed safely in selected patients. However, the absence of statistically significant predictors of complications and the modest sample size underscore the exploratory nature of these findings. Longer-term oncological outcomes and cost-effectiveness were not assessed and remain critical to determining the broader value of this approach. As robotic platforms continue to evolve, larger prospective and comparative studies will be required to clarify their advantages over conventional minimally invasive techniques, establish oncologic equivalence, and define the most appropriate role of robotic surgery in the management of CRC.
